# Predicting Old-age Mortality Using Principal Component Analysis: Results from a National Panel Survey in Korea

**DOI:** 10.3390/medicina56070360

**Published:** 2020-07-18

**Authors:** Jaeyong Shin, Kwang-Soo Lee, Jae-Hyun Kim

**Affiliations:** 1 Department of Policy Analysis and Management, College of Human Ecology, Cornell University, Ithaca, NY 14853, USA; drshin@ajou.ac.kr; 2 Department of Preventive Medicine, School of Medicine, Ajou University, Suwon, Gyeonggi-do 16499, Korea; 3 Department of Health Administration, College of Health Sciences, Yonsei University, Wonju, Gwangwondo 26493, Korea; planters@yonsei.ac.kr; 4 Department of Health Administration, College of Health Science, Dankook University, Cheonan-si, Chungcheongnam-do 31116, Korea

**Keywords:** artificial intelligence, AI, principal component analysis, forecast, mortality, older adult

## Abstract

*Background and Objectives:* This study aimed to group diseases classified by the International Classification of Diseases using principal component analysis, and discuss a systematic approach to reducing the preventable death rate from a perspective of public health. *Materials and Methods:* Using a 10-year follow-up analysis of the Korean Longitudinal Study of Aging (KLoSA) data, this study obtained de-identified data including participants’ data of community-dwelling individuals aged ≥45 years from 2006 to 2016. Participants were randomly selected using a multistage, stratified probability sampling based on geographical area and housing type. We excluded 37 participants with missing information at baseline and included 10,217 study participants. This study used the principal component analysis to extract comorbidity patterns, and chi-square test and Cox proportional hazards models for analyzing the association between the factors of interest. *Results:* Principal component 1 (diabetes, heart disease, and hypertension) was associated with an increased hazard ratio (HR) of 1.079 (95% confidence interval (CI) 1.031–1.129, *p* = 0.001). Principal component 3 (psychiatric and cerebrovascular diseases) was related to an increased HR of 1.134 (95% CI 1.094–1.175, *p* < 0.0001). Moreover, principal component 4 was associated with a high HR of 1.172 (95% CI 1.130–1.215, *p* < 0.0001). However, among participants aged between 45 and 64 years, principal component 4 showed a meaningfully increased HR of 1.262 (95% CI 1.184–1.346, *p* < 0.001). In this study, among the four principal components, three were statistically associated with increased mortality. *Conclusions:* The principal component analysis for predicting mortality may become a useful tool, and artificial intelligence (AI) will improve a value-based healthcare strategy, along with developing a clinical decision support model.

## 1. Introduction

Aging is a global phenomenon that is particularly observed in industrialized countries [1]. Presently, the proportion of older individuals in these countries is growing at an accelerated pace, mainly due to increasing longevity. Among the industrialized countries, South Korea shows the fastest aging society among the Organisation for Economic Co-operation and Development (OECD) countries, and the related health care cost has been growing [2]. The proportion of individuals aged ≥65 years was 7.2% in 2000 and 14.2% in 2017. In 2025, 20.3% of the total population in Korea will be aged ≥65 years. This aging speed is 7 years faster than that in Japan.

As the population is aging, it is important to estimate the population mortality based on medical conditions. Most estimation methods for mortality are based on a single independent factor (e.g., blood pressure, glycemic variability [3], muscle strength [4], self-rated health [5], health-rated quality of life [6], health literacy, and frailty). Otherwise, other estimators are calculated as a single score using weighted values to predict mortality. For example, Charlson et al. [7] defined numerous clinical conditions through reviewing hospital charts and assessed their relevance in the prediction of 1-year mortality. The Charlson Comorbidity Index (CCI) has been validated in large populations [8,9,10,11]. Similarly, the Elixhauser Comorbidity Index is also widely used to predict mortality [12].

However, since a systematic approach becomes a trend in medical research [13,14], it is questionable as to whether the comorbidity indices may have limitations in providing mortality prevention strategies based on the perspective of public health. In particular, members of the elderly population aged ≥65 years may suffer from multiple noncommunicable diseases. The multiple diseases share common risk factors, such as smoking, alcohol drinking, insufficient physical activity, and obesity [15,16]. Therefore, it is important to group the associated disease entities that may share some disease causes and pathophysiology using a machine-learning or econometric approach [16]. This study aimed to group each disease classified by the International Classification of Diseases Tenth Edition, using principal component analysis, and discuss the systematic approach to reduce preventable death rate from the perspective of public health.

## 2. Materials and Methods

### 2.1. Study Sample and Design

This study was conducted by the Korea Labor Institute for this rapidly growing population and obtained de-identified data from the first wave of the 2006 Korean Longitudinal Study of Aging (KLoSA), including participants’ data of community-dwelling Korean individuals aged ≥45 years until follow−up in 2016. Participants were randomly selected using a multistage, stratified probability sampling design based on geographical area and housing type across the country to create a nationally representative sample. As per the KLoSA protocol, trained surveyors obtained informed consent from participants and conducted face-to-face interviews using a computer-assisted personal interviewing program. The data were composed of seven categories, including population, family, health, employment, income, wealth, and subjective and life expectation. In the baseline survey in 2006, 10,254 individuals from 6171 households (1.7 per household) were interviewed.

To forecast the association between frequent comorbidities and all-cause mortality among these individuals, we excluded 37 participants with missing information at baseline 2006 and finally included 10,217 study participants. Thus, comorbidities for these participants were investigated, comorbidity patterns were derived from principal component analysis, and the association between each pattern and mortality was analyzed. This study doesn’t require ethical approval, because it is not a study using human derivatives, and all subjects are encrypted and cannot be identified.

### 2.2. Independent Variables 

With both questionnaires surveyed on the middle-aged and elderly panel and diseases that frequently develop in the elderly population, the diagnoses of hypertension, diabetes, malignant tumor, chronic lung disease, liver disease, heart disease, psychiatric disease, and arthritis or rheumatism; experience of falls in 2 years; and difficulty in daily activities due to visual impairment were analyzed to extract comorbidity patterns. Each comorbidity was categorized according to a “yes” or “no” response to the question “Have you ever been diagnosed by a doctor?”

### 2.3. Dependent Variable

Death (all-cause mortality) over a maximum follow-up period of 10 years was determined by a death certificate and coroner’s report.

### 2.4. Control Variables

#### 2.4.1. Socioeconomic and Demographic Factors

Age groups were divided into five categories: 45–54, 55–64, 65–74, and ≥75 years. Educational level was categorized into four groups: elementary school or lower, middle school, high school, and college or higher. Sex was categorized as male and female, and residential region was categorized into urban (administrative divisions of a city: Seoul, Daejeon, Daegu, Busan, Incheon, Kwangju, or Ulsan) or rural (not classified as administrative division of a city). Marital status was divided into two groups: single or married. The single group included separation and separation by death or divorce. Labor was divided into two categories: yes or no. Health insurance was categorized as National Health Insurance or Medical Aid.

#### 2.4.2. Health Status and Behavior Factors

Smoking status was categorized into three groups: current smoker, former smoker, or never smoker. Alcohol use was also divided into three groups: current drinker, former drinker, or never drinker. Moreover, the number of chronic diseases, such as hypertension, diabetes, cancer, chronic obstructive pulmonary disease, liver disease, cardiovascular disease, cerebrovascular disease, psychiatric disease, and arthritis, was also included as a covariate in our analyses.

### 2.5. Analytical Approach and Statistics

This study conducted a principal component analysis with varimax rotation, which is a statistical technique used at one level of principal component analysis to extract comorbidity patterns [17]. The principal component analysis analyzes the correlations between item and variables to identify principal components with high correlation. In this study, we included principal component scores by calculating a weighted sum of the items. Each item’s weight is derived from its principal component loading and each item’s contribution to the principal component score depends on how strongly it relates to the principal component. Because those weights are all between −1 and 1, the scale of the principal component scores will be very different from each other. Results of the principal component analysis indicated 4 categories of 11 items, which measure disease comorbidity patterns. In addition, chi-square test and Cox proportional hazards models were used to investigate the association between comorbidity patterns and all-cause mortality through a 10-year follow-up database. In all analyses, the criterion for statistical significance was a *p*-value of <0.05 in a two-tailed test. All analyses were conducted using the SAS software package version 9.4 (SAS Institute Inc., Cary, NC, USA)

## 3. Results

### 3.1. Sample Characteristics

**Figure 1:**
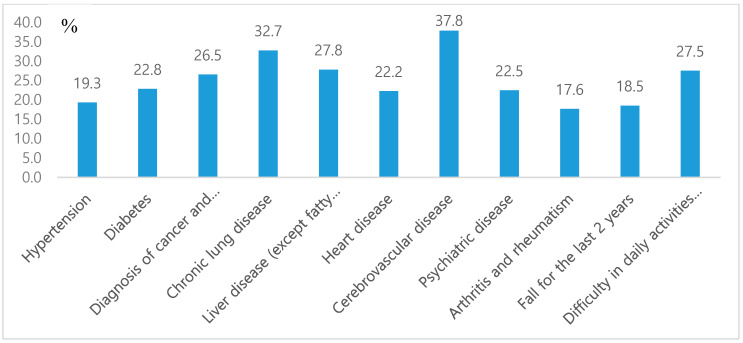
General characteristics for mortality by disease.

Table 1 presents the descriptive statistics of all variables at baseline (2006). In the 10,217 participants included in our study, the incidence of mortality was 1,487 (14.6%). In 2,828 participants (27.7%) with hypertension, the mortality rate was 19.3% (*n* = 546), and in 1,219 participants (11.9%) with diabetes, the mortality rate was 22.8% (*n* = 278). In 245 participants (2.4%) with malignant tumor, the mortality rate was 26.5% (*n* = 65), and in 226 participants (2.2%) with chronic lung disease, the mortality rate was 32.7% (*n* = 74). These two chronic medical conditions have the highest mortality rate among the included comorbidities (Figure 1).

In terms of sociodemographic factors, in 4,799 participants (47.0%) with an educational level of elementary school, the mortality rate was 21.8% (*n* = 1047); in 3,571 participants (35.0%) in the rural area, the mortality rate was 17.7% (*n* = 631); and in 2,281 participants (22.3%) living alone, the mortality rate was 24.8% (*n* = 24.8%). Participants with Medical Aid (*n* = 640, 6.3%) had a mortality rate of 25.2% (*n* = 161). In health behavior, former smokers (*n* = 976, 9.5%) and former drinkers (*n* = 687, 6.7%) had high mortality rates, with 23.2% (*n* = 226) and 27.8% (*n* = 191), respectively.

### 3.2. Principal Component Analysis for Grouping Chronic Medical Conditions

Table 2 shows the results of principal component analysis among 11 chronic medical conditions. As a result of calculating the Cronbach alpha coefficient through correlation analysis for each of the four principal components, it was confirmed that the four disease-oriented principal component domains were independent of each other at 0.00067. According to the result, we can identify four distinct principal components: principal component 1 (Disease of the circulatory system: diabetes, heart disease, and hypertension), principal component 2 (Disease of visual and musculoskeletal system: difficulty in daily activities due to visual impairment, arthritis and rheumatism, and fall during the last 2 years), principal component 3 (Disease of mental disorder: psychiatric and cerebrovascular diseases), and principal component 4 (Disease of the respiratory and digestive system: liver disease except fatty liver, diagnosis of malignant tumor, and chronic lung disease).

### 3.3. Association Between Grouped Principal Components and All-Cause Mortality

In Table 3, we performed a survival analysis to investigate the relationship between all-cause mortality and each grouped principal component. The presence of principal component 1 (diabetes, heart disease, and hypertension) was associated with an increased hazard ratio (HR) of 1.079 (95% CI 1.031–1.129, *p* = 0.001). The presence of principal component 3 (psychiatric and cerebrovascular diseases) was related to an increased HR of 1.134 (95% CI 1.094–1.175, *p* < 0.0001). Moreover, principal component 4 was associated with a high HR of 1.172 (95% CI 1.130–1.215, *p* < 0.0001). In participants aged between 45 and 64 years, principal component 4 (liver disease except fatty liver, diagnosis of malignant tumor, and chronic lung disease) showed a significantly increased HR of 1.262 (95% CI 1.184–1.346, *p* < 0.001). Other principal components were not significantly associated with an increased mortality rate. In participants aged ≥65 years, principal component 1 (HR = 1.065, 95% CI 1.012–1.120, *p* = 0.015), principal component 3 (HR = 1.140, 95% CI 1.096–1.187, *p* < 0.0001), and principal component 4 (HR = −1.132, 95% CI 1.083–1.184, *p* < 0.0001) were associated with an increased mortality rate. This section may be divided by subheadings. It should provide a concise and precise description of the experimental results, their interpretation as well as the experimental conclusions that can be drawn.

## 4. Discussion

In this study, we suggest that increased mortality rate is associated with principal component 3 (Disease of mental disorder: psychiatric and cerebrovascular diseases), principal component 1 (Disease of the circulatory system: diabetes, heart disease, and hypertension), and principal component 4 (Disease of the respiratory and digestive system: liver disease except fatty liver, diagnosis of malignant tumor, and chronic lung disease) in the elderly Korean population aged ≥65 years. Similarly, principal component 4 is associated with an increased mortality rate in the population aged between 45 and 64 years.

This result is similar to that in the national statistics of Korea. Among the population aged ≥65 years, malignant tumor has been the leading cause of death since 2007. Cardiovascular disease was the second leading cause of death in 2017. Respiratory disease is the fourth leading cause of death, and neurologic disease is the fifth. Additionally, other diseases comprising principal component 3 and 4 are among the most common causes of death. Although the Difficulty in daily activities due to sight in principal component 3 was excluded based on statistical criteria, it is known that it is closely related to brain disease and psychiatric disease [18]. Therefore, the result of the study is valid based on the crude national statistics.

In the population aged between 45 and 64 years, principal component 1 and 3 are not related to increased mortality, but the result was only marginally insignificant. The two principal component entities both have relatively long disease progression and manageable medical plans compared to principal component 4 entities. For example, liver disease was the third leading cause of death in the middle-aged population in 2017. Since the prevalence rate of hepatitis B and C in Korea is 0.8%, and in hepatitis C, interferon-γ treatment does not have a promising effect, with direct-acting oral regimens only being approved in Korea in 2016, the study population did not have sufficient treatment options during the study period. Without HCV screening, it may progress to liver cirrhosis or malignancy without specific symptoms.

This result is similar to that in the national statistics in Japan. The five leading causes of death in the Japanese population aged between 40 and 59 years are malignant neoplasm, heart disease, suicide, cardiovascular disease, and liver disease [19].

Through the valid mortality results from the principal component analysis, we may have meaningful implications in preventing premature mortality. First, we may consider group-based strategies in public health. Most prevention programs in Korea are based on a single disease or single risk factor approach (e.g., the Korean national diabetes prevention program [20,21] or national smoking cessation program [22,23]). In this study, we found that principal component 1, which includes diabetes, heart disease, and hypertension, is associated with increased mortality rate in individuals aged ≥65 years. These diseases share important risk factors and health behaviors, such as decreased daily activity [24], unbalanced diet [25,26], and smoking [26,27,28,29,30,31]. Therefore, it is necessary to control the group-based target diseases rather than a single-disease approach.

Although we know that it is important to prevent the risk from common and important factors in non-communicable diseases, it is difficult to have a scope that includes all factors. Thus, we suggest that the principal component analysis for mortality may have an effect on researchers and policy makers to review the common factors for prevention strategies in mortality. Second, policy makers in healthcare may have targeted strategies for preventing mortality based on the principal component analysis. However, we can determine which factor may have the largest population involved and the highest association with mortality. In this study, principal component 2 has the largest number of deaths (*n* = 602), followed by principal component 1 (*n* = 470), principal component 3 (*n* = 191), and principal component 4 (*n* = 184). However, principal component 2 does not have a statistically significant association with mortality. Therefore, it is assumed that principal component 1, which has the second largest number of deaths, has the largest impact on the Korean population. For principal component 1, which includes hypertension, diabetes, and heart disease, we have to implement an educational program for the general population who can be potential patients in principal component 1. In contrast, we have a different strategy for principal component 4. Although principal component 4 has the smallest number of deaths in the study, it has the highest mortality rate in the study population. Therefore, it is important to determine the risk group earlier and provide high-quality healthcare to prevent mortality in the principal component 4 group. Since we can estimate the number of deaths based on the factors and the degree of relationship to mortality through the principal component analysis, it will help policy makers to establish tailored strategies in preventing mortality. Lastly, we can follow the mortality based on the principal component analysis. The grouped principal components are different annually. In the 1970s, the most common cause of death in Korea was cerebrovascular disease, while it was malignancy in the 2010s [32,33]. This means that the causes of deaths have changeable dynamics and should be monitored annually. Using the principal component analysis, we can generate the grouped principal components and can have a proper health policy for preventing mortality. The dynamics also help us to understand the macroscopic health-related issues in Korea and how they can be managed using policy intervention.

In addition, we suggest that studies revealing multiple grouped conditions associated with mortality may improve a value-based healthcare strategy, along with the advanced estimation model using AI. Since 2019, the Centers for Medicare and Medicaid Services (CMS) has launched the projects for AI Health Outcomes Challenge and offered federal grants and contracts to innovators to demonstrate how AI tools—such as deep learning and neural networks—can be used to predict unplanned hospital and skilled nursing facility admissions as well as adverse events [34,35]. A variety of lifestyle and health data enable us to analyze negative health outcomes at once and prevent such harmful triggers as soon as possible. Other healthcare trends also explain tangible AI healthcare service model as well [36].

Through technology, we may provide mortality estimation service among the elderly population. It can be defined this process as AI-based decision support system for healthcare [37]. First, it is possible to calculate a probability or a propensity of mortality using the five principal components, those we have suggested using the health insurance claim data. Second, we can add their socio-demographic factors to increase the predictability of the model and capture unobservable factors related to mortality. It is also possible to obtain this from administrative data. Moreover, we can obtain their functional status using the biennial national health screening in Korea [38]. It contains information of lifestyle (e.g., smoking status, binge drinking, regular exercise, etc.), as well as functional status reflecting frailty among the elderly population (e.g., time to up and go, simple cognitive function test, etc.). Third, we can develop a clinical decision support model to analyze the probability of mortality risk. Finally, we can target the vulnerable population, which has shorter life expectancy than the same age group. Based on the result, we can perform a checkup with respect to more specific medical conditions in order to prevent early mortality cases through national screening. Therefore, we believe that our study can provide an essential step in the entire process of targeting the vulnerable population to increase the efficiency of the healthcare system, in other words, towards a value-based healthcare system [39].

This study has several limitations. First, medical history is self-reported. We believe that fatal diseases, such as malignancy or cerebrovascular disease, have low probability of ambiguity, but there is still a risk that participants may have biased memory or skip recording information. Second, the mortality cases may have duplicated and complex comorbidities. For example, more than half of the population aged ≥65 years have two or more noncommunicable diseases in Korea [40]. It is possible that two or more diseases may have an effect on mortality. Third, although we calculated the Cronbach alpha coefficient through correlation analysis, it was confirmed that the principal component scores for each principal component were independent of each other, but it still remains likely that there are multicollinearity problems. Fourth, the basic difference between principal component analysis and confirmatory factor analysis is the a priori assumption is that each factor is associated with a specified subset of indicator variables. The major limitation behind principal component analysis is its simplicity. Therefore, it is necessary to analyze the results through confirmatory factor analysis in a future study. Lastly, we investigated association rather than causality. Although we could measure the association between the factors and mortality, it is still possible that there are some unknown confounders.

## 5. Conclusions

We analyzed the association between diseases and mortality using principal component analysis. Among the four principal components, three are statistically significantly associated with increased mortality rate. The principal component analysis for grouping causes of death or disease categories can generate meaningful predictors for the analysis of mortality risk. This approach may become a useful tool for providing comprehensive and targeted strategies to healthcare professionals and policy makers. Further study can develop a clinical decision support model to analyze the probability of mortality risk along with advanced estimation model using AI.

## Figures and Tables

**Table 1:** General characteristics of subjects included for analysis.

	Total		Mortality				*p*−Value
	N	%	No	%	Yes	%	
Age							<0.0001
45–54	3288	32.18	3187	96.93	101	3.07	
55–64	2789	27.30	2580	92.51	209	7.49	
65–74	2671	26.14	2170	81.24	501	18.76	
≥75	1469	14.38	793	53.98	676	46.02	
Education							<0.0001
≤Elementary school	4799	46.97	3752	78.18	1047	21.82	
Middle school	1656	16.21	1500	90.58	156	9.42	
High school	2705	26.48	2504	92.57	201	7.43	
≥College	1057	10.35	974	92.15	83	7.85	
Gender							<0.0001
Male	4451	43.56	3657	82.16	794	17.84	
Female	5766	56.44	5073	87.98	693	12.02	
Residential Region							<0.0001
Urban	6646	65.05	5790	87.12	856	12.88	
Rural	3571	34.95	2940	82.33	631	17.67	
Marital Status							<0.0001
Married	7936	77.67	7014	88.38	922	11.62	
Single (including Separated, divorced)	2281	22.33	1716	75.23	565	24.77	
Labor							<0.0001
Yes	3950	38.66	3694	93.52	256	6.48	
No	6267	61.34	5036	80.36	1231	19.64	
National Health Insurance							<0.0001
Health insurance	9577	93.74	8251	86.15	1326	13.85	
Medical aid	640	6.26	479	74.84	161	25.16	
Smoking Status							<0.0001
Never	7274	71.20	6352	87.32	922	12.68	
Former smoker	976	9.55	750	76.84	226	23.16	
Smoker	1967	19.25	1628	82.77	339	17.23	
Alcohol Use							<0.0001
Never	3871	37.89	3397	87.76	474	12.24	
Former Drinker	687	6.72	496	72.20	191	27.80	
Drinker	5659	55.39	4837	85.47	822	14.53	
Total	10217	100.00	8730	85.45	1487	14.55	

**Table 2:** Results of principal component analysis.

	PC 1	PC 2	PC 3	PC 4
Cerebrovascular Disease	0.179	−0.118	**0.748**	0.006
Psychiatric Disease	−0.056	0.186	**0.734**	0.007
Difficulty in Daily Activities Due to Sight	0.218	**0.475**	0.182	0.069
Heart Disease	**0.365**	0.196	0.096	0.030
Hypertension	**0.707**	0.105	0.080	−0.004
Diagnosis of Cancer and Malignant Tumor (Excluding Slight Skin Cancer)	0.023	−0.080	0.031	**0.512**
Chronic Lung Disease	−0.180	0.397	0.024	**0.497**
Fall for the Last 2 Years	−0.060	**0.593**	0.014	−0.083
Liver Disease (Except Fatty Liver)	0.110	−0.065	−0.036	**0.711**
Diabetes	**0.729**	−0.047	−0.046	0.038
Arthritis and Rheumatism	0.269	**0.594**	−0.096	−0.079
Eigen Value	1.621	1.080	1.049	1.012

The highest principal component loadings of more than 0.3 are bold.

**Table 3:** Survival analysis based on the grouped principal components, based on the age group

	Mortality					
	Total		45-64		≥65	
	HR	*p*-Value	HR	*p*-Value	HR	*p*-Value
PC 1	1.079	0.001	1.097	0.096	1.065	0.015
PC 2	0.968	0.158	0.982	0.762	0.968	0.196
PC 3	1.134	<0.0001	1.088	0.051	1.140	<0.0001
PC 4	1.172	<0.0001	1.262	<0.0001	1.132	<0.0001
Age						
45–54	1.000		1.000		N/A	
55–64	1.847	<0.0001	1.690	<0.0001		
65–74	3.865	<0.0001	N/A		1.000	
≥75	10.278	<0.0001			2.751	<0.0001
Education (≥College)						
≤Elementary School	1.561	0.000	2.095	0.001	1.331	0.046
Middle School	1.133	0.365	1.444	0.126	0.994	0.972
High School	1.064	0.634	1.348	0.184	0.945	0.727
Gender (vs Female)						
Male	2.236	<0.0001	3.338	<0.0001	1.937	<0.0001
Residential Region (vs Urban)						
Rural	1.293	<0.0001	1.449	0.002	1.247	0.000
Marital Status (vs Married)						
Single (including Separated, Divorced)	1.491	<0.0001	1.930	<0.0001	1.327	0.000
Labor (vs No)						
Yes	0.577	<0.0001	0.486	<0.0001	0.646	<0.0001
National Health Insurance (vs Medical Aid)						
Health Insurance	0.899	0.215	0.775	0.223	0.938	0.499
Smoking Status (vs Never)						
Former Smoker	1.319	0.002	1.255	0.262	1.332	0.003
Smoker	1.460	<0.0001	1.302	0.108	1.458	<0.0001
Alcohol use (vs Never)						
Former Drinker	1.144	0.132	1.165	0.457	1.150	0.160
Drinker	1.149	0.047	1.179	0.264	1.149	0.083

HR: hazard ration, PC: principal component, N/A: not available.
